# Recent Advances in Nanoparticle-Based Drug Delivery Strategies to Cross the Blood–Brain Barrier in Targeted Treatment of Alzheimer’s Disease

**DOI:** 10.3390/pharmaceutics18020192

**Published:** 2026-02-01

**Authors:** Hoa Le, Giang T. T. Vu, Amos Abioye, Adeboye Adejare

**Affiliations:** 1 Faculty of Pharmaceutics and Pharmaceutical Technology, Hanoi University of Pharmacy, Hanoi 100000, Vietnam; 2 Department of Pharmaceutical Sciences, Philadelphia College of Pharmacy, Saint Joseph’s University, Philadelphia, PA 19104, USA; 3 School of Pharmacy, College of Pharmacy and Health Sciences, Belmont University, Nashville, TN 37212, USA

**Keywords:** blood–brain barrier, tight junctions, brain-targeting ligands, Alzheimer’s disease, amyloid-beta oligomer, amyloid-beta plaque, tau protein tangle, protofibrils, therapeutic nanoparticles, drug transport

## Abstract

The blood–brain barrier (BBB) is a major obstacle to the development of brain-targeted drug delivery systems, restricting greater than 98% of small molecules (<500 Da) and virtually all large-molecule drugs from entering the brain tissues from the bloodstream, resulting in suboptimal drug doses and therapeutic failure in the treatment of Alzheimer’s disease (AD). However, the advent of nanotechnology has provided significant solutions to the BBB challenges, enabling particle size reduction, enhanced drug solubility, reduced premature drug degradation, extended and sustained drug release, enhanced drug transport across the BBB, increased drug target specificity and enhanced therapeutic efficacy. In corollary, a library of brain-targeted surface-functionalized nanotherapeutics has been widely reported in the current literature. These promising in vitro, in vivo and pre-clinical results from the existing literature provide quantitative evidence for the relative clinical utility of each of the techniques, indicating remarkable capacity for brain-targeted carrier systems; many of them are still being tested in human clinical trials. However, despite the recorded research successes in drug transport across the BBB, there are currently no clinically proven medications that can slow or reverse the progression of AD because most of the novel therapeutics have not been successful during the clinical trials. Therefore, the main option for the treatment of AD is symptomatic treatment using cholinesterase inhibitors and N-methyl-D-aspartate (NMDA) receptor antagonists. Although these therapies help to alleviate symptoms of AD and improve patients’ quality of life, they neither slow the progression of disease nor cure it. Thus, an effective disease-modifying therapy for the treatment of AD is an unmet clinical need. It is apparent that a deeper understanding of the structural complexity and controlling dynamic functions of the BBB in tandem with a comprehensive elucidation of AD pathogenesis are crucial to the development of novel nanocarriers for the effective treatment of AD. Therefore, this narrative review describes the contextual analysis of several promising strategies that enhance brain-targeted drug delivery across the BBB in AD treatment and recent research efforts on two major AD biomarkers that have revolutionized AD diagnosis, amyloid-beta plaques and phosphorylated tau protein tangle, as potential targets in AD drug development. This has led to the Food and Drug Administration (FDA)’s approval of two intravenous (IV) anti-amyloid monoclonal antibodies, Lecanemab (Leqembi^®^) and Donanemab (Kisunla^®^), which were developed based on the Aβ cascade hypothesis for the treatment of early AD. This review also discusses the recent shift in the Aβ cascade hypothesis to Aβ oligomer (conformer), a soluble intermediate of Aβ, which is the most toxic mediator of AD and could be the most potent drug target in the future for a more accurate and effective drug development model for the treatment of AD. Furthermore, various promising nanoparticle-based drug carriers (therapeutic nanoparticles) that were developed from intensive research are discussed, including their clinical utility, challenges and prospects in the treatment of AD. Overall, it suffices to state that the advent of nanotechnology provided several innovative techniques for overcoming the BBB and improving drug delivery to the brain; however, their long-term biosafety is a relevant concern.

## 1. Introduction

**Figure 1:**
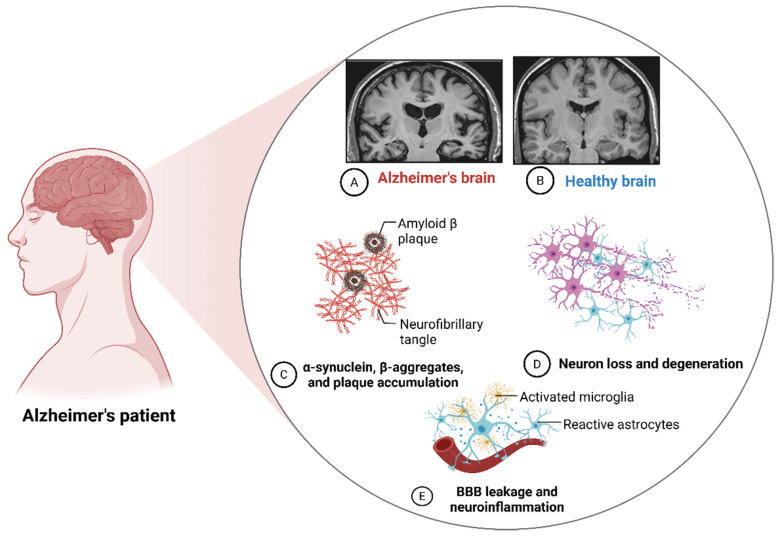
Schematic representation of the neuropathological features of Alzheimer’s disease: (**A**) is the scan image, showing the diffuse cortical atrophy of Alzheimer’s brain; (**B**) is a representation of normal healthy brain; (**C**) represents the accumulation of amyloid-beta plaque and neurofibrillary tangle of the phosphorylated tau protein; (**D**) represents neuronal degradation and (**E**) represents microglial activation and neuroinflammation. Created in BioRender. Abioye, A. (2025) https://BioRender.com/japq4ch.

Alzheimer’s disease (AD) is a chronic, irreversible and progressive neurodegenerative brain disorder that is characterized by brain shrinkage and gradual neuronal death, resulting in the progressive deterioration of brain function, memory loss, cognitive decline and behavioral changes that impact patients’ quality of life significantly [1]. Its clinical manifestation typically starts with a short-term memory impairment, followed by a progressive multifaceted cognitive syndrome that includes temporal–spatial disorientation, language deterioration, executive dysfunction and neuropsychiatric disturbances, as well as complete functional dependency at the terminal stages [1]. The initial clinical trajectory of AD is quite insidious, spanning several decades and evolving through three distinct phases: preclinical accumulation of amyloid-beta plaques and phosphorylated tau proteins, prodromal mild cognitive impairment, and dementia with irreversible neurological degeneration [1]. AD affects an estimated 7.2 million Americans aged 65 and above, which could increase to 13.8 million by 2060, and the current cost is about $345 billion annually, which is projected to increase to $1 trillion by 2050 if no new medication is discovered to prevent or treat AD [2]. Globally, AD is the most common type of dementia, affecting over 50 million people, which is projected to increase to 152 million by 2050 due to the accelerated aging of the global population. It has been suggested that one person develops dementia every three seconds, and the number of AD-related deaths is expected to increase from 2.38 million per year in 2019 to 5.8 million per year in 2050 [3]. According to the WHO’s report, the estimated global cost of dementia is expected to increase from US$1.3 trillion in 2019 to US$2.8 trillion by 2030 [4]. Thus, AD ranks as the fifth-leading cause of mortality in individuals aged 65 years and older, and it is the most prevalent neurodegenerative disease, causing severe global socioeconomic burdens on healthcare systems across several aging populations. Currently, there is no therapeutic intervention that can slow down or cure the disease. In AD, the accumulation of amyloid-beta (Aβ) in the brain leads to the formation of toxic protofibrils and senile plaques within the extracellular space, which has been shown to activate the disruption of the electron transport chain, leading to an increased production of reactive oxygen species (ROS) that can damage the mitochondria, leading to subsequent neuronal dysfunction, cognitive impairment and memory loss [5]. Similarly, protofibrils and plaques develop in the hippocampus region of the brain, which encodes memories, and the cerebral cortex, which is used for thought processing and decision making, resulting in neuronal damage or death as well as neurodegeneration and cognitive deficits. Thus, the brain changes from normal to an AD brain (Figure 1A,B). Several postmortem studies have revealed that AD is characterized by a pronounced extracellular deposition of amyloid-beta (Aβ) plagues and an intracellular accumulation of neurofibrillary tangles of hyperphosphorylated tau (τ) protein (Figure 1C), cholinergic neuronal death (Figure 1D), neuroinflammation (Figure 1E), and oxidative stress [6,7,8]. In the same vein, the increased activity of tau kinases in the brain can trigger the buildup of tau protein (Figure 1C), leading to the overproduction of misfolded or abnormal tau proteins, which can undergo self-polymerization and form aggregates known as neurofibrillary tangles (NFT) that cause neuronal damage and the subsequent memory loss [9,10].

The hallmarks of AD are the extracellular amyloid-beta aggregates and the intracellular tau protein aggregates, which can be measured in cerebrospinal fluid (CSF) or plasma. Thus, several molecular biomarkers like amyloid-beta isoform 42 (Ab42) and phosphorylated tau proteins have been detected in the CSF and used to identify patients’ AD status, particularly in clinical settings [11]. However, AD is a progressive neurodegenerative disease that causes gradual damage to the neurons in the brain without any clinical symptoms for at least 20 years, and the patient’s brain tissue is not available for a series of analyses to accurately monitor the progression of AD. The CSF biomarker screening is highly invasive; therefore, its use is limited [11]. Some less invasive brain-imaging techniques like positron emission tomography (PET) scans and magnetic resonance imaging (MRI) are well-established for the diagnosis of AD [12]. However, considering the heterogeneity of the AD patient population, a single biomarker is not adequate to fully characterize each patient. Therefore, several advanced techniques like omics technologies, artificial intelligence (AI) and machine learning (ML) tools have been deployed to improve the accuracy of biomarker-based testing, like the accurate electrical signal measurements of brain waves, electroencephalogram (EEG), AI-based language skills and memory tests, the quantification of molecular patterns, and computational analysis, which can resolve the heterogeneity of each patient [13,14,15]. Therefore, a deeper understanding of the crux of Alzheimer’s disease pathogenesis and the available treatment options is crucial to making a science-informed decision in developing appropriate medication for the treatment of AD.

## 2. Method of Review

The selection of articles for this review was based on key terminologies that are relevant to the objectives of the review, including blood–brain barrier, Alzheimer’s disease, brain-targeted drug delivery system, mechanism of drug transport across BBB, tight junctions, brain-targeting ligands, amyloid-beta oligomer, amyloid-beta plaque, tau phosphorylated protein tangle, protofibrils, nanoparticle-based drug delivery across BBB, therapeutic nanoparticles, and surface-functionalized nanoparticles. The articles were selected from articles published between 1980 and 2025 through scientific databases like ScienceDirect, Google Scholar, and PubMed, with particular attention given to recent advances in the diagnosis and treatment of Alzheimer’s disease, novel biomarkers of AD pathology, advanced therapeutic development for AD, preclinical and clinical trials, and prospects and challenges of nanotherapeutic carrier systems.

## 3. Challenges of Delivering Drugs Across the Blood–Brain Barrier (BBB)

**Figure 2:**
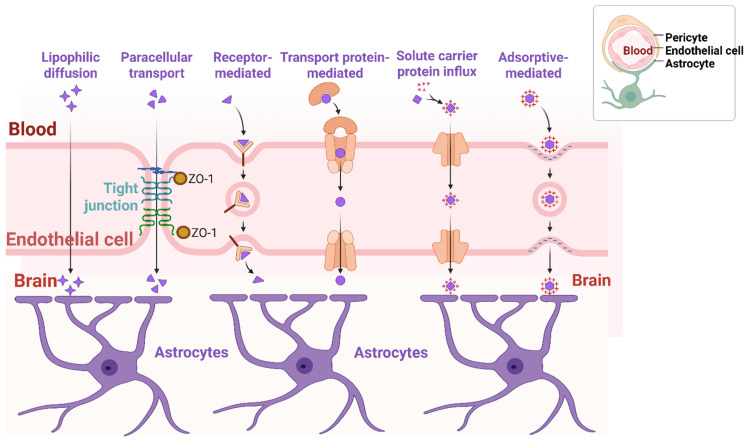
Schematic representation of the mechanisms of drug delivery across the blood–brain barrier (BBB). Created in BioRender. Abioye, A. (2025) https://BioRender.com/g2f60lt.

The BBB is a complex, highly specialized and dynamic structure that separates the blood circulation system from the brain parenchyma. It is essential for maintaining a stable environment for neuronal functions and protecting the brain from pathogens, toxins, and other harmful substances in the blood stream, thereby acting as a barrier to drug delivery to the brain [16,17]. Specifically, the BBB maintains homeostasis within the CNS, controlling the influx of nutrients and efflux of waste and toxic molecules, maintaining optimum ion concentrations, controlling immune surveillance, and separating central and peripheral neurotransmitter pools [18,19]. Structurally, it comprises endothelial cells, pericytes, astrocytes, the extracellular matrix and neighboring neurons (Figure 2), which work together and communicate through functional interaction and cell signaling [16]. Morphologically, the endothelial cells in the BBB do not have transcellular pores (also known as fenestrations or pinocytotic vesicles), and the adjacent endothelial cells are closely packed and uniquely fastened to each other, forming a continuous membrane barrier (tight junctions) that limits the diffusion of molecules between the blood circulation and the brain [20]. The tight junction is a highly occlusive paracellular or intramembrane barrier, comprising several transmembrane proteins like claudins, occludins and junction adhesion molecules (JAMs), as well as cytoplasmic proteins like zonula occludins (ZO), that seal adjacent endothelial cells controlling the paracellular transport of molecules, as illustrated in Figure 2 [21,22,23]. They are localized at the apical membrane of the brain endothelial cells, playing a crucial role in forming and maintaining the barrier function and cell polarity [24]. The tight junctions in brain endothelial cells also contribute to the high trans-endothelial electrical resistance of the BBB, enhancing the barrier function of the BBB by limiting the paracellular movement of charged particles. In the same vein, brain endothelial cells express a variety of substrate-specific transporters and receptors that form transport barriers on the luminal and/or abluminal membranes. They also express sets of CYP 450-metabolizing enzymes that act as metabolic barriers, including CYP1B1, CYP2U1 and CYP-3AF, which control the transport of nutrients, energy metabolites, and other essential molecules from the blood to the brain and the transport of waste products from the brain interstitial fluid to the blood [25]. These enzymes also play a significant role in metabolizing several drugs, including antiepileptic drugs [19]. Similarly, various efflux transporters act as transporter barriers, including P-glycoprotein (P-gp), breast cancer resistance protein (BCRP), and organic anion-transporting polypeptide (OATP), which may actively pump substances (including xenobiotics) out of the brain vascular endothelial cells back into the blood, resulting in decreased CNS exposure to the drug [25]. The structural integrity of the BBB is maintained by the astrocytes, pericytes and basal lamina (Figure 2). Astrocytes (astroglia) are the most widely expressed glial cells throughout the brain and are involved in stabilizing BBB, the perivascular clearance of wastes, regulating blood flow, balancing the neuroimmune responses within the CNS and providing the link between the neurons and the brain vasculature through their end-feet [19]. The pericytes are located in the basement membrane on the abluminal side of the endothelial cells and are involved in maintaining BBB’s functionality through paracrine signaling and physical interactions, while the basal lamina is an extracellular matrix protein [26].

Overall, the complex microcellular architecture of the BBB confers selective permeability to allow molecules with specific characteristics to pass through, as depicted in Figure 2. Thus, the BBB is a major obstacle to the development of disease-modifying therapeutics for AD, restricting the passage of >98% of small drug molecules (<500 Da) and approximately 100% of large drug molecules (>500 Da), including monoclonal antibodies (mAbs) and antisense oligonucleotides (ASOs), which are about 150 kDa and 7 kDa respectively [27,28,29]. For example, small lipophilic molecules like heroin and anesthetics with molecular weights less than 500 Da can enter the brain by passive diffusion through transcellular or paracellular routes, while larger lipophilic molecules greater than 500 Da like proteins, peptides, monoclonal antibodies and nanoconjugates do not cross the BBB [16]. Other small hydrophilic molecules like amino acids, electrolytes and nucleosides, as well as large hydrophilic drugs like hormones, insulin, iron and lipoproteins, may require active transport like carrier-mediated transcytosis, adsorptive-mediated transcytosis, receptor-mediated transcytosis, and cell-mediated transcytosis. This poses a great challenge for the delivery of disease-modifying monoclonal antibodies (mAbs), as only about 0.1% of the mAbs dose administered has been reported to cross the BBB [30]. Even the very small amount of the drug that crosses the BBB is subjected to the ATP-binding cassette (ABC) transporters in the luminal and abluminal region of the endothelial cell, which may actively pump the drug back into the systemic circulation [31]. Therefore, several potential mAbs have failed in clinical trials because they do not cross the BBB in sufficient quantities to produce a therapeutic effect [32]. Although the BBB selectively allows essential nutrients, ions, oxygen, hormones and glucose that are required for healthy brain function to cross the barrier into the brain while allowing the movement of metabolic waste products out of the brain, it limits the permeability of harmful molecules, including most CNS drug candidates. However, in spite of the strict restriction by the BBB, the literature is replete with the fact that nanoparticle-based drug delivery systems can utilize the intrinsically specialized BBB transport mechanisms to transport drugs into the targeted sites in the brain, particularly the receptor-mediated transcytosis (RMT) and adsorptive-mediated transcytosis (AMT) [33,34,35]. This targeted approach of using therapeutic nanoparticles is particularly critical for the precise delivery of therapeutic agents to amyloid plaques and inflamed brain tissues, maximizing drug efficacy and minimizing systemic side effects in the treatment of AD. Therefore, a deeper understanding of the structural complexity and dynamic controlling functions of the BBB and the underlying pathogenicity of Alzheimer’s disease is crucial for the rational design of drug nanocarriers that can not only get beyond the BBB and reach the brain parenchyma but also present sufficient bioavailability for enhanced therapeutic efficacy in the treatment of Alzheimer’s disease. Overall, the distinctive features of the BBB provide sturdy protection for the brain by limiting the entry of chemical substances into the brain, making it difficult to deliver therapeutic agents to the brain for the treatment of neurodegenerative diseases, including Alzheimer’s disease and brain cancer. In corollary, several studies in the current literature have reported great successes in enhancing drug transport across the BBB, and a library of brain-targeted surface functionalized nanotherapeutics is widely reported. However, most of the results are from in vitro, in vivo and pre-clinical evaluations, and some of them that entered human clinical trials have not been very successful. Therefore, the focus of this review is to explore novel nanoparticle-based delivery strategies that can penetrate, bypass or modify the BBB to facilitate efficient drug delivery to their targets in the brain and enhance therapeutic efficacy in AD treatment.

## 4. Advances in Current Treatment Options for Alzheimer’s Disease (AD)

Presently, there are no clinically proven medications that can slow down or reverse the progression of AD because of its complex, heterogeneous and long-term (chronic) nature [36]. Therefore, the main option is symptomatic treatment using cholinesterase inhibitors like donepezil (Aricept^®^), rivastigmine (Excelon^®^) and galantamine (Razadyne^®^) because they have demonstrated modest efficacy in alleviating symptoms of AD and play a key role in cognitive function [37,38]. For example, the effect sizes for cognitive scores in patients administered with donepezil 10 mg/day (one dose), galantamine 24 mg/day (two doses) and rivastigmine 6–12 mg/day (2 doses) relative to placebo ranged from 0.27 to 0.49, indicating a small to medium magnitude of improvement in cognitive functions (efficacy) in AD [39]. In a similar study, Winblad et al. demonstrated cognitive benefits of rivastigmine transdermal patch versus capsules on AD patients. They concluded that 10 cm^2^ dose of rivastigmine patch provided superior safety profile and similar efficacy to the highest doses of the capsule dosage form [38]. It is apparent that the accurate and timely detection of AD biomarkers like amyloid-beta and phosphorylated tau in cerebrospinal fluid are a valuable diagnostic insight to engender personalized treatment that can halt disease progression and improve the quality of life of these patients. However, biomarker confirmation in AD has offered little or no clinical utility due to its invasive nature and prohibitive cost. Also, there are no treatment options even if the diagnosis is confirmed, as most of the available treatment options are for symptomatic relief. In the recent past, the US Food and Drug Administration (FDA) approved five therapeutic agents for the symptomatic treatment of AD, including cholinesterase inhibitors like tacrine in 1993 [40], donepezil in 1996 [41], rivastigmine in 2000 [42] and galantamine in 2001 [43], and N-methyl-D-aspartate (NMDA) receptor antagonists like memantine and a combination of memantine with donepezil [44]. Since neurodegeneration in AD results in the depletion of cholinergic neurotransmitter (acetylcholine) levels in the brain, it suffices to state that inhibiting the enzymes that break down the neurotransmitters is a potential therapeutic strategy to restore neurotransmitter levels, enhancing neuronal survival. For example, donepezil and galantamine are acetylcholinesterase inhibitors (AChEIs), which inhibit the activity of acetylcholinesterase (AChE), thus preventing the degradation of acetylcholine in synaptic gaps, amplifying the cholinergic effects, maintaining neuronal activity, and enhancing memory and learning abilities [45]. In the same vein, rivastigmine increases the concentration of acetylcholine in the brain by blocking acetylcholinesterase (AChE) and butyrylcholinesterase (BuChE), thereby improving communication between neurons [45]. In contrast, memantine, an uncompetitive NMDA receptor antagonist, reduces the excitatory amino acid system-related neurotoxicity and neuronal apoptosis in the synapse [45], thereby protecting the brain against excessive levels of the neurotransmitters like glutamate. Although cholinesterase inhibitors exhibited symptomatic efficacy in a double-blind, randomized controlled clinical trial, their efficacy on cognitive function was significantly low and they did not affect amyloid-beta or tau pathology [46]. Despite these available therapeutic options, the challenge of attaining the required drug bioavailability in the brain persists because of the BBB and the tandem efflux transporters that pump drug molecules against the concentration gradient back into the bloodstream, thus limiting the drug efficacy [47]. Thus, higher drug doses, greater than the minimum effective concentration, are required in the brain, leading to severe side effects like nausea, vomiting and headache that often lead to the discontinuation of treatment. Also, most of these drugs have short biological half-lives, requiring multiple frequencies of administration per day, resulting in wasted doses, erratic absorption, poor pharmacokinetics and pharmacodynamics, therapeutic failure, severe side effects, and non-adherence to dosage regimen. For example, galantamine and rivastigmine exhibit half-lives of 7 and 2 h respectively [37,48]. Recently, the FDA and European Medicines Agency (EMA) gave expedited approval for two disease-modifying anti-amyloid-β humanized IgG-1 monoclonal antibodies targeting the aggregated beta-amyloid species aducanumab (Aduhelm^®^) and lecanemab (Leqembi^®^) that can remove Aβ plaques from the brain, thus reducing cognitive and functional deterioration at the early stage of AD, including in patients with moderate cognitive dysfunction or mild dementia and elevated levels of Aβ in the brain [49,50]. However, aducanumab was discontinued by the manufacturer in November 2024 due to concerns about its effectiveness in reducing cognitive decline in AD patients [49]. A random clinical trial data (NCT04437511) has shown that donanemab (Kisunla^®^) significantly inhibited cognitive decline among participants with an early stage of AD, including amyloid and tau pathology, suggesting a comparative effectiveness to lecanemab [51]. Therefore, lecanemab (Leqembi^®^) and donanemab (Kisunla^®^) are the two available intravenous (IV) infusion monoclonal antibodies (mAbs) that remove amyloid-beta from the brain in patients with early AD [52]. In the same vein, intensive research efforts are ongoing on the antisense oligonucleotides (ASOs) and other small molecule drugs [53,54]. These are significant scientific advancements and breakthroughs in addressing the complexities of treating Alzheimer’s disease by enabling the microglia-mediated phagocytosis of protofibrils and plaques respectively; however, they present significant side effects, requiring careful therapeutic monitoring. Also, they do not cure AD and are not suitable for all types of AD cases due to the heterogeneity of AD treatment response, disease progression, phenotypic variability and risk factor susceptibility. Thus, longer clinical trials are required to determine the effectiveness and safety of these monoclonal antibodies. Although significant progress has been made in drug transport across the BBB, it is apparent that the ability to cross the BBB is necessary but not sufficient for therapeutic efficacy in the disease progression-focused treatment of AD because it exhibits multifactorial and complex network-driven pathological processes. It is important to note that the current evidence is not sufficiently robust to confirm any long-term impact of these new medications on AD patients’ behavior, cognitive function and lifestyles. It suffices to state that no drug is currently available to modify or slow the progression of AD, and the current standard treatments with the four drugs (donepezil, galantamine, rivastigmine, and memantine) are not effective in reducing cognitive decline in clinical settings [36,55]. Thus, the challenges for the effective treatment of AD remain an unmet clinical need. Although BBB is the primary barrier between the systemic circulation and the brain, preventing more than 98% of small molecules and approximately 100% of biologics from reaching the brain tissues [56], the advent of nanotechnology has revolutionized brain-targeted drug delivery through the BBB. It is envisioned that novel therapeutic nanoparticle (NP) carrier systems will enhance AD treatment effectiveness by improving the bioavailability, pharmacokinetics and pharmacodynamics of the therapeutic agents, and reduce potential side effects. Therefore, the focus of this review article is to explore the recent advances in nanoparticle-based drug delivery strategies that can overcome the BBB obstacle and enhance the targeted treatment of AD simultaneously.

## 5. Current Strategies for Brain-Targeted Drug Delivery in Alzheimer’s Disease

### 5.1. BBB Modulation to Facilitate Therapeutic Uptake in Alzheimer’s Disease

Neurological disorders are commonly associated with BBB disruption and a loss of integrity, which can alter permeability to several molecules due to BBB leakage and the possible entry of the therapeutic molecules into the brain. Thus, the AD pathology-induced modification of the BBB may significantly influence therapeutic transport across the BBB [16]; however, the impact may vary with different neurological diseases. For example, BBB’s dysfunction in AD leads to impaired BBB transporter function, resulting in reduced clearance and the subsequent accumulation of amyloid-beta (Aβ) plaques and the resulting neurodegeneration. In the same vein, the neurofibrillary tangles (NFTs) formed by the hyperphosphorylated tau protein (pTau) can disrupt the neuronal microtubule, leading to neuronal toxicity and death. Similarly, ischemic stroke has been reported to disrupt BBB, enhancing its permeability in two phases—early disruption within 4–6 h and delayed disruption after 2–3 days—which could constitute a therapeutic advantage for controlled drug release [57]. In multiple sclerosis, BBB disruption facilitates a significant entry of immune cells into the brain, leading to autoimmune reactions and epileptic seizure [58]. Also, the BBB leakage could cause fluid accumulation in the perivascular spaces; thus, blood products and cellular debris could enter the enlarged perivascular spaces, preventing the entry of large therapeutic molecules [59]. Several factors like heterogeneous amyloid pathology, variable vascular damage and neuroinflammation may influence the integrity of the BBB, resulting in dysfunction and varying permeability, but it is not clear if the alteration of the BBB integrity enhances or impedes the transport of therapeutic molecules into the brain [59]. The intra-arterial injection of hyperosmotic agents like mannitol has been utilized to enhance permeability by increasing the pore size of the BBB tight junction; however, long-term exposure may cause an accumulation of neurotoxins. Therefore, the temporary opening of the BBB tight junctions in targeted areas was explored using focused ultrasound (FUS) waves with microbubbles that can induce temporary and reversible disruptions in the BBB. This type of opening was reported to enhance transcellular drug transport and influence the expression of ABC transporter [60]. Recent studies have demonstrated that the FUS-induced generation of temporary and reversible openings or channels in the endothelial cells with a high level of accuracy provides some form of gateway into the brain parenchyma, which is a promising strategy for brain-targeted drug delivery [28]. Preclinical AD models in rodents and non-human primates have demonstrated the effectiveness of FUS in reducing AD pathology, even in the absence of therapeutic intervention [61,62]. Although, the mechanism of FUS-induced amyloid reduction is not fully understood, it has been proposed that BBB opening by FUS promotes the activation of microglial cell followed by the internalization of the amyloid plaque [61]. In the same vein, unilateral BBB opening in transgenic AD tau mouse models has resulted in bilateral reduction in phosphorylated tau [63]. In a human phase I safety clinical trial, Lipsman et al. utilized magnetic resonance-guided FUS combined with IV-injected microbubbles to open the BBB temporarily, reversibly and repeatedly in five AD patients [64]. They concluded that FUS-induced BBB opening is safe, as it did not cause any serious clinical or radiographic adverse events nor any significant worsening of cognitive score for three months compared to baseline. It is apparent that FUS-induced amyloid removal is beneficial in AD animal models; however, the repeated activation of microglia can result in neuroinflammation and a subsequent exacerbation of AD in human patients. Therefore, further research is urgently needed to elucidate the mechanism of FUS-induced amyloid reduction and the impact of repeated treatments in AD patients.

### 5.2. Mechanisms of Therapeutic Nanoparticle Transport Across the BBB in Alzheimer’s Disease

#### 5.2.1. Passive Diffusion

Passive or lipophilic diffusion is triggered by a concentration gradient, which facilitates the spontaneous movement of drug molecules from a region of high concentration to a region of low concentration without using energy, as depicted in Figure 2. Factors that may influence the passive diffusion of NPs across the BBB include the lipophilicity of the drug molecule, particle size, surface charge, and hydrophilic/lipophilic balance. Lipophilic drug molecules with small particle size and spherical shape tend to exhibit increased diffusion rate and permeability across the BBB compared to large and elongated molecules because of their preferential solubility in the lipid bilayer and the ability of spherical molecules to navigate narrow intercellular gaps [65]. However, the transport of lipophilic molecules may be restricted by efflux transporters such as P-glycoprotein (P-gp), which are extensively expressed on the luminal region of the BBB endothelial cells, pumping out the therapeutic NPs, thus decreasing the absorption of the drug into the brain tissues. In contrast, hydrophilic or charged molecules will not permeate the BBB readily. Similarly, the presence of specific functional groups like aromatic ring can increase BBB permeability due to hydrophobic interaction with the endothelial cells [66], while the presence of polar functional groups can hinder passive diffusion across the BBB [67]. Therefore, a deeper understanding of the mechanism of passive diffusion is critical to rational drug design and the prediction of brain-targeted drug delivery systems in AD. Several advanced techniques like computer modeling, structure–activity relationship studies and in vivo–in vitro correlation models have been utilized to evaluate and predict the efficiency of passive diffusion [68].

#### 5.2.2. Adsorptive-Mediated Transcytosis

**Figure 3:**
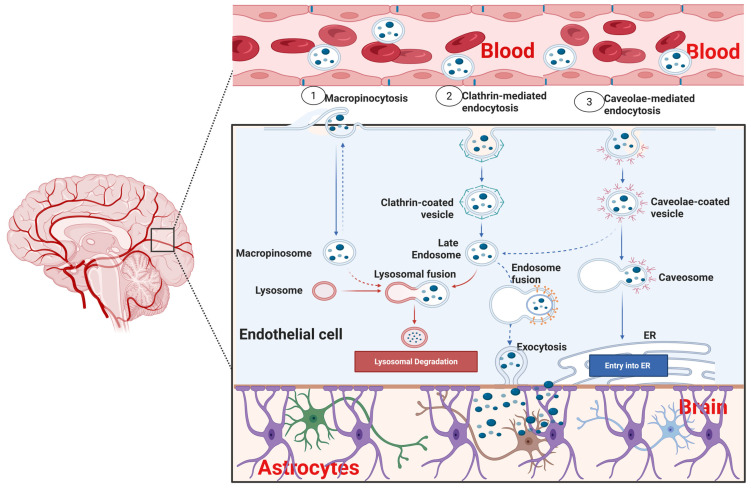
Schematic representation of the clathrin- and caveolae-mediated transcytosis. Created in BioRender. Abioye, A. (2026) https://BioRender.com/c7uayoo.

Therapeutic nanoparticle uptake across the BBB requires specialized techniques that can exploit the unique properties of the BBB endothelial cell and utilize its specific cellular transport mechanisms or bypass them. Adsorptive-mediated transcytosis (AMT) is triggered by the electrostatic interaction between a positively charged NP cargo and the negatively charged heparan sulfate proteoglycans residue on the luminal surface of the endothelial cell membranes [33,34]. This interaction facilitates the non-specific endocytosis of the NPs by the endothelial cells through the clathrin- or caveolin-independent pathways [69]. The internalized NPs are transported across the cell and released into the site of action in the brain parenchyma. Thus, the surface modification of the therapeutic NPs to carry a positive charge using cationic polymers like chitosan and polyethylenimine (PEI) can increase NP uptake efficiency via AMT. However, excessive cationic charge density may be toxic; thus, a careful optimization of the process would be of great value. Notably, the engulfment of NP cargoes inside the caveolae at the luminal or apical side of the endothelial cell does not degrade the cargo during the intracellular transport; however, the contents are degraded within the lysosomes, as depicted in Figure 3.

#### 5.2.3. Receptor-Mediated Transcytosis

In contrast to the AMT, receptor-mediated transcytosis (RMT) involves a controlled and selective binding of therapeutic NPs in the blood stream to specific transmembrane receptors expressed on the luminal side of the BBB endothelial cells, including transferrin receptor (TfR), insulin receptor (IR), nicotinic acetylcholine receptor (nAChR), etc. [70]. Through this energy-dependent intuitive interplay between therapeutic NPs and ligands, drug molecules undergo adsorption, transcytosis across the BBB and a subsequent release into the brain parenchyma with relatively high efficiency. This involves decorating the nanoparticles with specific ligands on their surface for targeting a particular receptor and thus promoting receptor-mediated endocytosis, which is triggered by the binding of the iron transport protein transferrin to the TfR. Thus, the bound ligand and receptor invaginate within the cell to produce an endosome that is transported across the cell, where the vesicle cargo is exocytosed and delivered to the brain parenchyma, reaching the neurons and the glia cells within the brain [71,72,73]. Ligand-functionalized NPs are able to bind to these receptors, internalized through the clathrin-mediated endocytosis and subsequently trafficked in clathrin-coated vesicles across the endothelial cell [35]. RMT is considered the most promising method and is widely employed for the brain delivery of nanoparticles because of its specialized characteristics, including high specificity, selectivity, and affinity. Several functionalized NPs have been studied for brain uptake efficiency, including NPs functionalized with transferrin, antibodies and peptides through the endogenous transferrin transport system [74]. Notably, the literature is replete with several BBB-RMT studies using transferrin receptor (TfR) for brain-targeted drug delivery. For example, insulin receptors are highly expressed in the brain, including the endothelial cells, hypothalamus, olfactory bulbs, hippocampus, striatum, cerebral cortex and cerebellum [75]. In contrast to transferrin, insulin has a short half-life in blood serum, which restricts its use as a brain-targeting agent. Nonetheless, insulin monoclonal antibodies have been used as brain-targeting ligands to recognize insulin receptors [76]. Several preclinical studies have shown that some antibodies are bispecific, binding both amyloid and TfR, thus increasing the antibody brain uptake by 7–50-fold in AD transgenic mice [77,78]. However, the expected results of amyloid reduction are inconclusive, as some studies demonstrated a decrease in the total number of amyloid plaques and soluble amyloid protofibrils [79,80], while other studies concluded that there was no effect on soluble amyloid protofibrils [81]. Nonetheless, the preliminary outcome of an ongoing phase IIa clinical trial of Trontinemab^®^ (Gantenerumab fused to a monovalent anti-human TfR1-binding Fab fragment) demonstrated rapid reduction in amyloid at lower doses compared to other anti-amyloid mAbs [82], which is quite promising. However, it is important to note that TfR1 is also expressed in other parts of the body like spleen, bone marrow and red blood cells, which may lead to potentially serious off-target adverse effects.

#### 5.2.4. Transporter-Mediated Transcytosis

Targeting the BBB-specific transporters is another effective way for therapeutic NPs to reach the brain tissue. The transporter-mediated transcytosis (TMT) mechanism enables the passage of several molecules across the BBB barrier by utilizing certain transport proteins that control the selective absorption and efflux of endogenous molecules, nutrients, drugs and other materials that are essential for the brain function [83]. Typically, each transporter protein family, including glucose transporters (GLUTs), amino acid transporters (AATs) and nucleoside transporters (NTs), have a unique substrate specificity and functional characteristics. For example, glucose transporter 1 (GLUT1) is the primary glucose transporter in the BBB, ensuring a constant supply of glucose for the brain’s energy needs [84], while AATs transport essential amino acids like leucine, isoleucine, valine, etc., across the BBB, facilitating absorption into the brain parenchyma [85,86]. The expression and functionality of these transporter proteins determine the absorption and disposition of the therapeutic drug in the brain and may be influenced by the state of disease, physiological circumstance and drug interaction. Thus, to develop an effective brain-targeted therapeutic nanoparticle and improve drug distribution in the brain, several research efforts have been made to understand the critical role of TMT in the cellular functions of the BBB, particularly in designing better brain-targeted drug delivery systems, polymer–drug conjugate design, prodrug design and transporter ligand development [87,88]. Therefore, a deeper understanding of the selectivity and transport abilities of several transporter proteins can be utilized to improve drug absorption into the brain parenchyma, enhancing the therapeutic efficacy of the brain-targeted drugs, particularly in the management of Alzheimer’s disease.

#### 5.2.5. Bypassing the BBB Through Intranasal Route

Several therapeutic nanoparticles have been investigated for the delivery of drugs to the brain through the nasal cavity because of the ease of administration, faster onset of action, ability to bypass the first-pass metabolism, and reduced degradation tendency. They can also bypass the BBB to reach the central nervous system (CNS) directly through olfactory and trigeminal pathways [89]. The olfactory pathway involves the absorption of the therapeutic NPs in the olfactory epithelium of the superior turbinate in the nasal cavity, followed by intracellular transport along the olfactory nerves through the cribriform plate to the olfactory bulb, then followed by intraneuronal transport within the olfactory neuron and extracellular diffusion along the perineural channels into the brain parenchyma in the hippocampus and other regions of the brain [90]. On the other hand, in the trigeminal nerve pathway, the therapeutic NPs are absorbed in the nasal cavity and transported directly to the brain stem through the trigeminal nerve fibers [89]. Recent studies on the brain-targeted intranasal delivery of insulin have demonstrated improved insulin signaling receptor activities and cerebral glucose metabolism in the hippocampus, enhancing neuronal function and cognition as well as memory restoration in AD patients [91]. Although brain-targeted intranasal drug delivery is a novel approach, it has several challenges, including a small surface area (150 cm^2^) for drug absorption, mucus secretion by goblet cells (which may trap the drug), mucociliary clearance by the ciliated cells (which can facilitate drug clearance) and enzyme degradation of drugs. Overall, the efficiency of this route is less than 1% [92]

### 5.3. Nanoparticles as Brain-Targeted Therapeutic Carriers in AD

**Figure 4:**
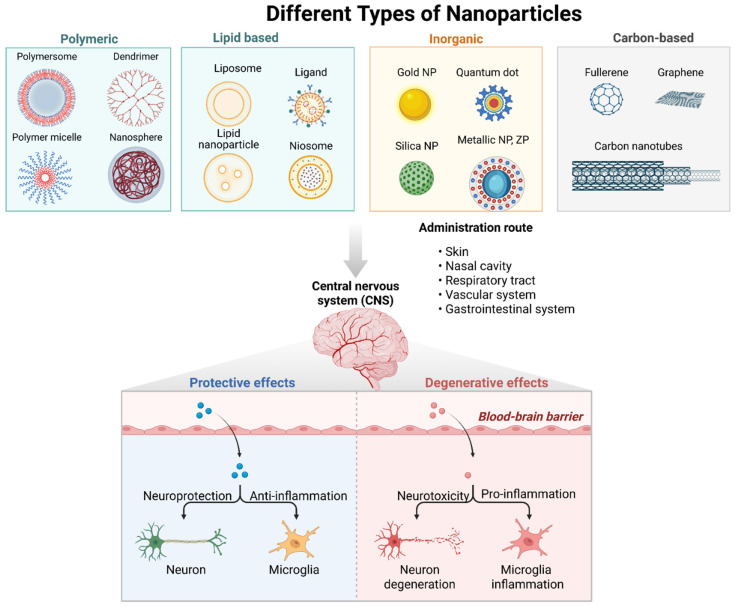
Schematic representation of the different nanoparticle-based brain-targeted therapeutic delivery systems for the treatment of Alzheimer’s disease. Created in BioRender. Abioye, A. (2025) https://BioRender.com/mlry3xk.

The development of novel brain-targeted chemical entities is quite challenging, rigorous and very expensive, and only about 3–5% of the drugs in the development pipelines have reached the market because most of them cannot cross the BBB in vivo [87]. However, using novel nanoparticle-based rational drug design may provide innovative solutions to this challenge. Nanoparticles are small particulate systems with dimensions of 1–1000 nm; in particular, sizes ranging from 1 to 100 nm exhibit uniquely ideal physicochemical properties for biomedical applications, including large surface area, customizable characteristics, extendable loading capacity (payloads) and brain-targeted drug delivery by crossing the BBB [93]. They are versatile drug delivery systems because they can mimic biological molecules like proteins [94], interacting with cells and tissues, and their physicochemical characteristics such as size, shape, hydrophobicity and surface charge (zeta potential) can be easily optimized to facilitate BBB penetration and therapeutic efficacy [93]. Their structure and surface characteristics can be precisely modified using a variety of formulation techniques to enhance drug solubility, protect therapeutic cargo from degradation and immune attacks, enhance BBB penetration efficiency, enhance permeability and retention (EPR) effect, extend the stability in the systemic circulation, control the drug release into specific target, enhance encapsulation efficiency and drug loading capacity at higher drug concentrations, and escape the reticuloendothelial system. Therapeutic NP surface can also be functionalized with targeting ligands such as amino acids, endogenous proteins, antibodies and peptides to enhance the BBB penetration by facilitating receptor-mediated transcytosis and other mechanisms with enhanced brain-target specificity, reducing off-target effects and achieving higher drug concentration at the site of action while reducing unwanted side effects [95]. Thus, they can traverse inaccessible targets like the brain and deliver therapeutic agents efficiently, enhancing the prospects of therapeutic success in the management of AD. However, the characteristic small size of nanoparticles does not confer an automatic crossing of the BBB, but the higher concentrations and large surface area increase the propensity for crossing the BBB [16]. The efficiency of BBB penetration by the nanocarriers depends on the interplay between the physicochemical characteristics of the polymeric nanocarrier and the endocytic mechanism of transport utilized by the BBB. Therefore, the rational design of nanotherapeutics by incorporating drug molecules into polymeric- or lipid-based nanoparticles could increase drug solubility, extend circulation half-life, facilitate targeted drug delivery and enhance therapeutic efficacy significantly [96]. Several nanoparticle-based therapeutic carriers have been developed to overcome the BBB obstacle, including polymeric nanoparticles, lipid-based nanoparticles, inorganic nanoparticles and carbon-based nanotubes, as depicted in Table 1 and Figure 4.

#### 5.3.1. Polymeric Nanoparticles

**Figure 5:**
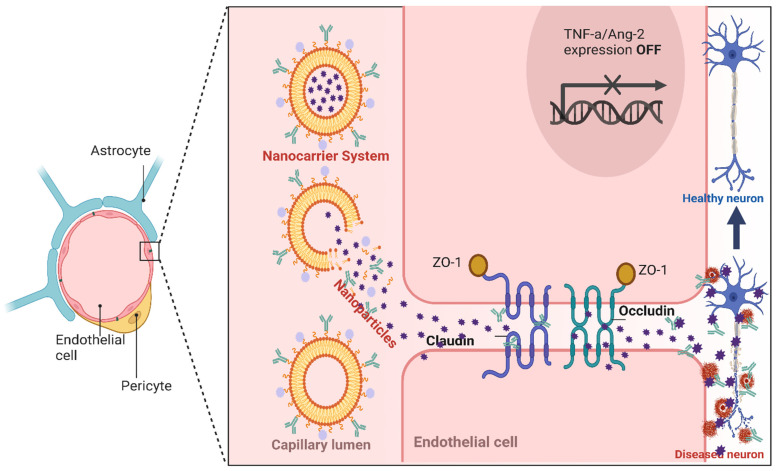
Schematic representation of the therapeutic NP transport across endothelial junction in the blood–brain barrier (BBB). Created in BioRender. Abioye, A. (2025). https://BioRender.com/lg9jv39.

Polymeric nanoparticles are colloidal dispersions of nanospheres, nanocapsules, nanoplexes or nanoconjugates in which the active drug is uniformly dispersed and covalently linked or adsorbed onto the surface of the carrier in a polymer matrix or incorporated into the inner core of a core–shell nanostructure. They are prepared with natural or synthetic biodegradable and biocompatible polymers such as chitosan, polylactic-co-glycolic acid) (PLGA), poly(ε-caprolactone) (PCL), etc., using several techniques, including the polymerization of a wide variety of monomers [119], ionic gelation or coacervation [120], spontaneous emulsification or solvent diffusion [121], emulsion solvent evaporation [122], nanoprecipitation [123], spray drying [124], and supercritical fluid technology [125]. Polymeric NPs are characterized by nano-range particle sizes between 1 and 100 nm, which facilitates bioaccumulation in tumor tissues through the enhanced permeability and retention (EPR) effect, enhanced drug loading efficiency, controlled drug release, extended systemic circulation time and reduced systemic toxicity [126]. The enhanced transport of NPs across the BBB can be explained by the bioaccumulation and retention of NPs in the brain blood capillaries, as well as the NPs’ adsorption on the capillary walls, setting up a high concentration gradient, which enhances drug transport across the BBB into the brain [127]. Therapeutic nanoparticles are commonly derived from natural, synthetic and hybrid polymeric materials; however, semi-synthetic and synthetic polymers such as chitosan and poly(lactic-co-glycolic acid) (PLGA) respectively are often preferred for AD medications [128]. Of particular interest is the ability of polysorbate 80-coated NPs to inhibit efflux system and increase the fluidity of the endothelial cell membrane, thus enhancing the drug permeability across the BBB. However, this may cause local toxicity, resulting in the intuitively reduced permeabilization of the brain endothelial membrane [128]. The polymeric nanoparticles can also permeate the BBB through the tight junctions, as depicted in Figure 5. Positively charged NPs can attract the negative surface charges of the endothelial membrane of the BBB, leading to an electrostatic interaction that facilitates the endocytosis of the NPs across the BBB [129,130]. However, NPs with high charge density (positive or negative) can undergo opsonization and systemic clearance [129,131]. To enhance the compatibility, circulation time and therapeutic efficacy of the nanoencapsulated drug, the detection of the therapeutic NPs may be concealed (protected) from microphages’ opsonization and systemic clearance by modifying the NPs’ surface using the cell membrane. Wang et al. demonstrated less macrophage-mediated phagocytosis in the NPs with RBC interface and a 2.9-fold increase in the uptake of PLGA NPs cloaked with RBC membrane NPs in the atherosclerotic plaques compared to the control NPs [132,133]. Wilson et al. also developed polysorbate 80-coated poly(n-butylcyanoacrylate) NPs for the brain-targeted delivery of rivastigmine for the treatment of AD [134]. The authors concluded that there was significant increase in rivastigmine uptake in the brain (*p* < 0.001) from the coated NPs compared to the uncoated NPs or the free drug. In a similar study, Joshi et al. utilized modified nanoprecipitation and emulsion polymerization techniques to prepare rivastigmine-loaded poly(lactic-co-glycolic acid) (PLGA) and poly(butylcyanoacrylate) (PBCA) nanoparticles respectively [135]. The control formulation in normal saline did not produce any noticeable improvement in learning and memory capacities, whereas the rivastigmine-loaded NPs exhibited a significant decrease in escape latency (*p*, 0.05), suggesting a reversal (antagonist) of the scopolamine-induced amnesia in the test groups. In the same vein, Fazil et al. formulated rivastigmine-loaded chitosan NPs using an ionic gelation technique [136]. The authors reported enhanced uptake and bioavailability of rivastigmine in the brain when administered through the intranasal route compared to the intravenous (IV) route.

Polyethylene glycol (PEG) is a commonly used polymer to develop NPs with longer circulating time in the blood stream, providing near-neutral surfaces, hydrophilicity, high biocompatibility and good tolerance. The tight association between ethylene glycol units and water molecules provides a highly hydrophilic environment, preventing nanoparticles from interacting with the hydrophobic domains of proteins, hindering opsonization and the mononuclear phagocyte system (MPS) clearance, and prolonging systemic circulation time [137]. The hydrating PEG layer stabilizes steric repulsion and shields the nanoparticle surface from aggregation, enhancing nanoparticle dispersion in water [138]. Therefore, PEG coating on the nanoparticle surface (PEGylation) can prevent their aggregation, opsonization, and phagocytosis and reduce NP cytotoxicity, prolonging the systemic circulation time, which subsequently increases the interaction opportunity between nanoparticles and brain vascular endothelial cells [139,140,141]. The PEGylation of nanoparticles alone does not allow them to cross the BBB; however, due to its improved blood circulation time, the probability of PEGylated NPs interacting with BBB receptors increases, leading to the enhanced brain accumulation of NPs. Several studies have shown that decorating NPs with PEG prevented protein adsorption and NP clearance, prolonging the blood circulation time and consequently enhancing PEGylated NPs’ brain uptake to a more significant extent than plain NPs [142,143]. Due to their unique physicochemical characteristics, advances in nanoparticle (NP) surface modifications such as drug–polyethylene glycol conjugation (PEGylation) and ligand conjugation have shown remarkable multifunctional abilities to cross the BBB by opening the tight junctions between endothelial cells, leading to a localized permeabilization of the BBB, allowing the penetration of the therapeutic agent as a free drug or NP–drug conjugate, as depicted in Figure 4 [144,145]. In this case, a transporter protein could be attached to a drug molecule to facilitate its transport across the BBB by specialized BBB transport mechanisms like receptor-mediated transcytosis (RMT), adsorptive-mediated transcytosis (AMT), transporter-mediated transcytosis, and endocytosis, releasing their contents into the cytoplasm, followed by exocytosis in the endothelium abluminal region. In the same vein, the therapeutic agent can be loaded into or conjugated with nanoparticle-based carriers like polymeric nanoparticles, liposomes, nanoconjugates, extracellular vesicles, etc., to enhance BBB permeability. Also, changing the route of administration to the intranasal route could deliver the drug directly to the brain parenchyma through the olfactory and trigeminal pathways, bypassing the BBB [16]. Overall, it is important to note that the PEG molecular weight, surface density, and conformation can significantly affect the protein opsonization on the PEGylated nanoparticles and the phagocytosis and MPS clearance, thereby affecting the stealth effect of nanoparticles in the body.

#### 5.3.2. Lipid Nanoparticles

Lipid-based nanoparticles are organic nanocarriers that contain fats, oil and related substances, including liposomes [146], niosomes [147], transferosomes [148], ethosomes [149], solid lipid nanoparticles [150] and nanostructured lipid carriers [151,152]. Liposomes are spherical therapeutic nanoparticles with a hydrophilic core for carrying drug molecules and an outer coating of lipid bilayer, as depicted in Figure 3. Drugs are loaded in the internal phase of the nanostructure to enhance the solubility of the active drug and protect it from premature degradation, and their sizes range between 50 and 500 nm, depending on the organic solvent used in their preparation and the ionic strength of the suspension medium [153]. Liposomes can be readily functionalized to facilitate BBB crossing and enhance drug-targeting and pharmacokinetic profiles, and have been used successfully to increase the effectiveness of approved medications for the treatment of AD including galantamine [154], rivastigmine [155], and donepezil [156]. Niosomes are vesicles that can be used as liposome substitute and are typically prepared by combining cholesterol and alkyl or dialkyl polyglycerol ether-derived non-ionic surfactants to produce lamellar nanostructures with particle sizes ranging from 10 to 100 nm (small unilamellar vesicles), 100 to 3000 nm (large unilamellar vesicles) and greater than 5 µm (multilayer vesicles). They are biocompatible, biodegradable and non-immunogenic, with longer circulation time and enhanced capability to cross the BBB, and have been successfully utilized in the intranasal delivery of rivastigmine for the treatment of AD [157,158]. Transferosomes are highly flexible and elastic lipid-based vesicles that can deform and pass through spaces that are 5–10 times smaller than their diameter, which is often less than 300 nm [159]. Thus, their high drug loading capacity and deeper layer penetration ability make them promising therapeutic carriers for the treatment of AD [160]. Their ultra-deformable characteristics have been ascribed to water evaporation, which occurs upon topical application, creating an osmotic gradient that facilitates the hydration of the dermis [161]. Ethosomes are also lipid-based nanocarriers, comprising phospholipids, ethanol and water, giving them unique skin penetration power and enhanced permeability. Recently, an ethosome comprising ethanol and phosphatidylcholine was developed for the encapsulation and transdermal delivery of ligustrazine for the treatment of AD [162]. However, further research is ongoing to determine the clinical utility of ethosomes in the management of AD. Solid lipid nanoparticles (SLNs) are lipid-based nanocarriers containing solid lipids with large surface area that are prepared using a high-pressure homogenizer without requiring any organic solvent [101]. SLNs can carry hydrophilic and lipophilic drugs, prevent premature drug degradation, maintain drug stability, increase drug retention in the systemic circulation, create a high concentration gradient of the drug to open tight junctions [163] and cross the BBB through receptor-mediated transcytosis and adsorptive-mediated transcytosis mechanisms [101]. Currently, SLNs are being utilized as advanced therapeutic delivery systems to provide sustained drug release, enhancing the bioavailability and therapeutic efficacy of several existing drugs that are used in the treatment of AD [164]. The nanostructured lipid carriers (NLCs) are the second-generation lipid nanoparticles (LNPs) that offer certain advantages to address the challenges of other LNPs, including high physical stability and encapsulation efficiency, low toxicity, and the ability to carry lipophilic and hydrophilic drugs with minimal surfactant concentration [151]. NLCs have been reported to enhance the bioavailability of drugs used in the treatment of AD [165,166].

#### 5.3.3. Inorganic Nanoparticles

Inorganic NPs are considered hydrophilic, biocompatible, non-toxic and highly stable in physiological conditions. They exhibit suitable physicochemical characteristics, including intrinsic surface plasmon resonance and electrical properties of metallic inorganic NPs, magnetic properties and optical properties [167,168]. Several metallic nanoparticles have been utilized extensively as therapeutic nanocarriers, including gold, iron oxide, gold–iron oxide nanocomplex, silver, platinum, cerium oxide, and mesoporous silica and silicon dioxide nanoparticles for the treatment of neurodegenerative diseases including AD [169]. They are easy to fabricate, surface-functionalize and conjugate with various biomolecules, including small-molecule drugs, DNA/RNA, antibodies, peptides and aptamers; thus, they are very versatile [167]. Gold nanoparticles (AuNPs) are essentially preferred because of their biocompatibility, high stability, ease of fabrication, significant potential for surface modification, size-dependent optical properties and Food and Drug Administration (FDA) approval [167].

#### 5.3.4. Carbon-Based Nanoparticles

Carbon nanotubes (CNTs) are tubular nanostructures comprising hexagonal networks of carbon atoms in the form of graphene sheets that are fused into a cylindrical shape [170]. Their unique physicochemical characteristics like low toxicity, minute particle size, electrical conductivity, mechanical strength, large surface area and ability to penetrate the BBB make them promising therapeutic nanocarrier systems for brain-targeted drug delivery [171,172]. Several recent studies on single-walled carbon nanotubes (SWCNTs) and multi-walled carbon nanotubes (MWCNTs) have demonstrated the potential of carbon-based nanoparticles in drug delivery to the brain. For instance, Yang et al. demonstrated that SWCNTs successfully delivered acetylcholine into the brain parenchyma for the treatment of chemically induced Alzheimer’s disease [173].

## 6. General Discussion and Current Challenges

Alzheimer’s disease (AD) constitutes a significant therapeutic challenge because of its multifaceted nature, involving a complex interplay between heterogenous pathogenesis and cognitive decline. The complex pathological processes include Aβ accumulation and clearance imbalance, tau protein hyperphosphorylation and neurofibrillary tangles, neuroinflammation, neuronal loss, and synaptic dysfunction. For example, a drug that crosses the BBB and targets only amyloid-beta plaque may not be clinically effective because it does not affect any of the other downstream pathologies. Thus, multifunctional drugs with therapeutic potential to simultaneously modulate several AD pathologies, enhance cognitive function, and promote Aβ clearance would provide a more effective treatment outcome. However, the limited understanding of the disease pathology and progression, inefficient drug localization at the site of action, poor drug bioavailability and pharmacokinetics, inadequate target interaction despite BBB penetration, insufficient intracellular drug concentrations, ineffective binding to the pathological oligomer, and lack of efficient technologies to deliver therapeutic agents to the target sites have led to disappointing results in clinical trials with little or no clinical translation of the preclinical findings. One of the key functions of the BBB is active efflux, moving several molecules out of the brain to prevent any buildup, thereby preserving brain homeostasis and normal brain function. Several efflux transporters like P-glycoprotein (P-gp), multidrug resistance-associated proteins (MRPs) and breast cancer resistance proteins (BCRPs) are predominantly expressed on the luminal side of the BBB endothelial cells to carry out the active efflux functions [174]. Thus, BBB is a significant primary obstacle to brain-targeted drug delivery systems, restricting greater than 98% of small molecules (<500 Da) and virtually all large-molecule drugs from entering the brain tissues [29]. However, therapeutic nanoparticles < 200 nm exhibit clinical promise and the ability to cross the BBB via enhanced permeability and retention (EPR) effect, thus providing critical opportunities to explore strategies that can enhance therapeutic efficacy with limited side effects. It is apparent that exploring clinically effective disease-modifying therapeutics for AD is one of the greatest challenges in the development of brain-targeted drug delivery systems, particularly due to the fact that causative factors of AD pathology (biomarkers) are poorly understood, and the subtle but complex nature of AD pathology evolves over several decades without any symptoms [175]. The onset of AD has been associated with the release of Aβ in the extracellular space, where it accumulates to form larger insoluble senile plaques composed primarily of Aβ and the abnormal aggregation of tau protein into neurofibrillary tangles, which hinder the formation of critical neuronal connections required for memory recollection, leading to the degeneration of neurons and the consequent lack of synapse processing [176]. Thus, Aβ plaques and tau protein tangles are considered significant biomarkers for AD pathology, and the current focus of therapeutic intervention for AD includes amyloid-beta (Aβ) targeting to inhibit the amyloid pathway, preventing its production and aggregation or eliminating the Aβ peptides that facilitate AD pathogenesis.

Therefore, for a long period of time, the therapeutic development paradigms have posited extracellular Aβ aggregates and intraneuronal hyperphosphorylated tau pathology as the cardinal hallmarks in AD pathogenesis. However, despite the crucial insights into Aβ neuropathology and metabolism, the clinical translation of the amyloid cascade hypothesis has been unexpectedly limited because several anti-amyloid therapies exhibited little or no clinical efficacy [177]. This underscores critical knowledge gaps in AD pathogenesis; thus, there is a recent shift in the Aβ cascade hypothesis from a focus on Aβ plaques and tau protein tangles as the causative agent for AD pathology to the small soluble Aβ oligomer intermediate, which is the most toxic mediator of AD, with the notion that targeting the most toxic oligomers may be more effective; this has been shown to protect cognition in rodent models [178]. Erten-Lyons et al. reported that the plaque burden does not correspond to cognition or neuron degeneration because there are non-demented individuals with advanced AD neuropathology, indicating that those individuals had significant plaque burden but no memory impairments or changes in brain volume [177]. In a similar study, insoluble Aβ plaques did not induce memory impairment in the absence of the intermediate Aβ oligomers in mice, and a reduction in oligomer levels improved memories in the mice, while soluble Aβ oligomers showed cognitive deficits in the absence of plaques [179,180]. In corollary, the soluble Aβ oligomer has been associated with initiating neuroinflammatory processes and possibly neuronal death, which has been ignored for over ten years [181]. In the same vein, it is apparent that the heterogeneity of AD pathogenesis and complexity of BBB’s structure and function are not fully understood, which may account for the limited or no success in the search for a cure for AD. For example, emerging research evidence indicates that BBB breakdown is both a contributor to and consequence of AD pathogenesis, creating self-perpetuating neurodegenerative loops via impaired Aβ clearance, inflammatory responses and neurodegeneration [59]. Thus, the current treatment regimen for AD mainly targets symptomatic relief rather than disease-modifying therapies. Recently, the FDA approved two disease-modifying anti-β amyloid humanized IgG-1 monoclonal antibodies [lecanemab (Leqembi^®^) and donanemab (Kisunla^®^)] targeting the aggregated Aβ senile plaque and tau fibrillary tangles [50]. These significant scientific advancements addressed some of the complexities in treating Alzheimer’s disease; however, they present significant side effects, requiring careful therapeutic monitoring. Despite several extensive research efforts in this area, none of the small-molecule anti-amyloid drug candidates for the treatment of AD have reached the market. Thus, presently, there is no cure for AD. Recently, the emergence of nanotechnology-enabled drug delivery designs has transformed the utility of nanoparticle-based carriers for brain-targeted drug delivery in the treatment of AD, which is quite promising. Most of them can cross the BBB, target specific sites of action and utilize multi-drug payload within a single delivery system, such that multiple sites are targeted simultaneously [133]. Additionally, the surface modification of the therapeutic NPs prolongs drug release, enables specific Aβ-targeting, increases NPs residence time in the systemic circulation, enhances therapeutic efficacy and reduces side effects. The higher circulation time has been ascribed to the hydrophilicity of the NP surface due to PEGylation, which reduces opsonization and subsequent clearance by the mononuclear phagocyte system (MPS) [133]. However, many of the research efforts are in vitro, ex vivo and pre-clinical studies; thus, further studies are required to evaluate the safety concerns and to validate the therapeutic effectiveness of nanotherapeutics in clinical trials, including desirable pharmacokinetics and pharmacodynamic profiles. In the same vein, concerns about their toxicity, safety and immunogenicity are considerable challenges that limit the clinical utility of NPs. Immunogenicity is a critical safety concern in AD, as poorly formulated NPs may activate the innate immune system, resulting in the release of proinflammatory cytokines and the activation of microglia and the complement system, which can further exacerbate neuronal degeneration [182]. For example, nanoparticles can readily cross the BBB and other biological membranes, causing potential mitochondrial dysfunction, inflammation, generation of reactive oxygen species (ROS) and oxidative stress [183]. They may also interact directly with circulating erythrocytes, leading to platelet aggregation, blood clotting or hemolysis, which may be fatal [184]. The literature is replete with studies demonstrating that PEGylation minimizes interaction with erythrocytes and effectively reduces the hemotoxicity of NPs [137,138,140,141,142], while the surface modification and functionalization of nanotherapeutics enhance drug-targeting efficiency, BBB penetration, and controlled drug release, preventing nonspecific drug interaction [133,185]. Thus, the physicochemical properties of the therapeutic NPs like particle size and zeta potential (surface charge) must be optimized to enhance their drug-loading capacity, encapsulation efficiency, controlled drug release profiles, therapeutic efficacy and minimized systemic toxicity. High loading capacity enables therapeutic NPs to carry a higher quantity of drug, which is crucial for crossing the BBB and optimal therapeutic efficacy [28]. Also, therapeutic nanoparticles have demonstrated promise as nanocarriers for the intranasal route of drug administration through the olfactory and trigeminal pathways, which physically bypass the BBB. Overall, the success rate of developing brain-targeted drugs is the lowest compared to other therapeutic routes and it takes longer to develop them than non-CNS therapeutics. Thus, creative and highly adaptable approaches, including nanotechnology-enabled rational drug design, are urgently needed for the expedited development of brain-targeted and clinically effective nanotherapeutics for the treatment of Alzheimer’s disease.

## 7. Conclusions and Future Perspectives

The AD therapeutic development pipeline is currently facing numerous challenges, and one of the major frustrations is that clinical endpoints in human clinical trials do not always reflect the in vitro molecular successes of promising nanotherapeutics. Several research efforts have produced novel drug molecules with remarkable ability to cross the BBB, including nanoparticles, nanoparticle carriers, surface-modified nanoparticles, surface-functionalized nanoparticles, etc. However, there are several controversial questions about whether nanoparticles could cross the BBB by themselves or are actively targeted to the brain by functionalized nanoparticles. Also, it is not clear how the heterogeneity of AD pathology impacts the efficiency of drug delivery and targeting of nanoparticle-based carriers through the BBB. Recent advances in nanotherapeutic strategies are quite promising, as most NPs < 200 nm can not only cross the BBB, particularly through the EPR effect in a damaged or leaky BBB and actively target a specific site of action, but can also utilize multi-drug payload within a single delivery system such that multiple sites are targeted simultaneously. It suffices to state that nanoparticle-based drug delivery systems have evolved as a robust therapeutic strategy for the future treatment of neurodegenerative diseases, particularly AD. However, a wider application of nanotechnology has been limited by some drawbacks, including the toxicity of the NPs, the lack of reproducible effects, potential scalability issues, NP–tissue interaction, and the cost of production. Also, patients’ heterogenicity and AD progression rate are poorly understood and may influence the drug delivery and distribution in the brain, requiring personalized treatment. Thus, the development and optimization of a scalable formulation of personalized nanomedicines using a Quality by Design (QbD) approach and quantum-enabled technologies would be of great value for patient adherence and enhanced therapeutic outcomes. Quantum-enabled technologies could be utilized to understand the tunable physicochemical and intrinsic critical attributes of the NP carriers, including their surface characteristics, polymer–drug conjugation, binding affinity, bond strength, drug-loading capacity, stability, supramolecular interaction, self-aggregation, self-assembly and functionality at the molecular level. In the same vein, Grover’s search in quantum computing algorithms could be useful tools for identifying and amplifying the toxic Aβ conformers, which can provide a unique platform for the rational formulation of novel and more effective Aβ conformer-targeted nanomedicines for the future treatment of neurodegenerative disorders like AD. Also, a thorough understanding of AD pathology and the rational development of stimuli-sensitive therapeutic NP carriers would be required to achieve precise brain-targeted drug release. In corollary, long-term safety concerns like hemotoxicity, immunogenic response, biocompatibility and bioaccumulation can be addressed through in vitro and in vivo toxicological profiling models. From the foregoing, it is apparent that advanced technologies like quantum computing, machine learning and pharmacogenomics would be of great value for the design and evaluation of stimuli-sensitive therapeutic nanocarriers that can identify, amplify and specifically target the toxic oligomers (conformers) in the treatment of Alzheimer’s disease.

## Figures and Tables

**Table 1:** Therapeutic nanoparticle-based brain-targeted delivery systems for Alzheimer’s disease.

Class of Nanoparticles	Delivery System	Therapeutic Agent	Ligand/Targeting Agent	Route of Administration	Mechanism of Drug Release	Therapeutic Outcomes	Limitation	References
Lipid-based nanoparticles	Liposomes [phospholipid bilayer]	Rivastigmine, donepezil, caffeic acid, galantamine, curcumin	ApoE 3, Transferrin, PEG, cell penetrating peptides	Intravenous (IV), intranasal	Passive diffusion, ligand-mediated targeting, bilayer destabilization.	Improved BBB crossing; enhanced drug stability; increased drug concentration in the brain tissue and extended circulation time; improved cognition and reduced Aβ in preclinical models.	Physical and kinetic instability like aggregation, drug leakage, poor drug-loading capacity (payload). Scale-up challenges. Lack of precise target localization, thus requiring surface functionalization.	[33,97,98,99,100]
	Solid-lipid nanoparticles (SLNs) [solid lipid core + surfactant]	Donepezil, quercetin, erythropoetin, curcumin, quercetin, galantamine, Ferulic acid, tarenflurbil	Transferin; lactoferin; surface modification using chitosan; PEG	IV, intranasal, oral	Sustained and extended release from solid-lipid matrix, drug targeting, prolonged drug release; enhanced BBB penetration, suitable for topical use.	Increased brain uptake, reduced oxidative stress and Aβ aggregation; enhanced brain bioavailability (preclinical).	Low drug-loading capacity, solvent-related toxicity, non-uniform particle sizes.	[99,101,102]
	Nanostructured lipid carriers (NLCs) [solid + liquid lipid matrix]	Rivastigmine, curcumin, berberine		IV, intranasal	Controlled release via disordered lipid matrix; improved BBB penetration; enhanced bioavailability.	Increased drug bioavailability in the brain and reduced AD pathology, including neuroinflammation.	Physical instability; prone to premature drug degradation; formulation is quite complex; long-term storage challenges; requires optimization of the surface characteristics.	[102]
	Polymeric micelles	Anti-miRNA-21 antisense oligonucleotide, curcumin, rapamycin	Lactoferrin, peptides	IV	Solubilization of hydrophobic drugs, controlled drug release.	Efficient BBB penetration, enhanced brain bioavailability, encapsulation and solubilization of hydrophobic drugs. Controlled delivery of anticancer drugs.	Long metabolic time; lower physical stability; premature drug release.	[103]
Polymeric nanocarriers	PLGA, chitosan, and PEG-based nanoparticles	Dopamine, beberine, diindolyl-methane, tacrine, paclitaxel, β-sheet breaker peptide	Tet-1 peptide, PEG, antibodies, lactoferin	IV, intranasal	Controlled biodegradation of polymer matrix. Slow release & absorption; reduce ROS; low toxicity; extended circulation time; increased drug circulation period.	Enhanced BBB transport and drug delivery to the brain, decreased amyloid burden (preclinical).	Inflammatory response, potential cytotoxicity from degradation products, scale-up challenges.	[104,105,106]
Inorganic nanoparticles	Mesoporous silica nanoparticles (MSNPs)	Curcumin, multifunctional cargoes	Surface peptides, targeting sequences	IV	pH- or surface-triggered controlled release from pores; high drug-loading capacity; easily dissolved DDS; chemical modification of gold surface can minimize toxicity.	High drug-loading capacity, controlled release, targeted drug delivery potential.	Safety and macrophage clearance concerns, still in early preclinical stages.	[107,108]
	Metallic nanoparticles (gold, iron oxide)	Imaging agents, therapeutic cagoes (xanthoceraside)	Transferrin, lactoferring	IV	Heat/trigger-enabled drug release (magnetic/photothermal).	Promising theranostic (diagnostic & therapeutic) effects in preclinical studies.	Accumulation tendency and long-term toxicity.	[109,110]
Dendrimers	PANAM, PPI dendrimers	Small molecule drugs, nucleic acids, carbamazepine, piperine minocycline	Multiple functional groups (COOH, NH_2_) for drug-targeting, TPGS lipopolysaccaride	IV, intranasal	Surface-facilitated drug release; enzyme-triggered response.	High drug-loading (payload) capacity, enhanced BBB crossing potential, extended drug circulation time, easily dissolved DDS. Improved brain uptake.	Risk of toxicity and immunogenicity, complex fabrication techniques. Acid- and alkaline-sensitive.	[111,112,113]
Biomimetic or cell-derived nanoparticles	Exosomes, cell membrane-coated nanoparticles	Endogenous proteins, drug molecules	Membrane receptor mimicry	IV, intranasal	Natural fusion and receptor interaction.	High BBB penetration, low immunogenicity.	Difficult large-scale preparation.	[33]
Carbon-based organic nanoparticles	Carbon nanotubes (CNTs), e.g., single-walled carbon nanotubes (SWCNTs) and multi-walled carbon nanotubes (MWCNTs)	Donepezil, curcumin, β-sheet breaker peptides, siRNA	PEGylation, transferrin, antibodies, amino-functionalization, rabies virus glycoprotein (RVG) peptide	IV, intranasal (preclinical)	Adsorption and desorption, redox-responsive or pH-triggered release, endocytosis-mediated intracellular release.	Efficient BBB penetration, inhibition of Aβ aggregation, improved neuronal uptake, neuroprotective effect in preclinical studies. Promising nanocarrier for brain-targeted drug delivery; high sensitivity; significant stability.	Potential neurotoxicity, oxidative stress, long-term accumulation and biodegradability concerns.	[114,115]
	Graphene oxide (GO)	Curcumin, quercetin, anti-Aβ peptides	PEG, lactoferrin, peptide ligands	IV, intranasal	Surface-mediated adsorption, sustained drug release, redox-responsive detachment.	Reduced amyloid fibrillation, antioxidant and anti-inflammatory effects, enhanced drug stability. Striatal targeting; high drug loading capacity; prolonged drug release; low toxicity; high biocompatibility.	Inflammatory response, hemocompatibility issues, dose-dependent cytotoxicity. In vivo studies are limited.	[116,117,118]
	Fullerenes (C60 derivatives)	Antioxidants, neuroprotective agents	Hydroxyl or carboxyl functionalization	IV (preclinical)	Passive diffusion and ROS-responsive drug release.	Enhanced antioxidant activity, protection against ROS-induced neuronal damage.	Poor solubility in aqueous and organic solvents. Limited targeting specificity. Functionalization required.	[117]

## Data Availability

No new data were created or analyzed in this study. No new data were created or analyzed in this study. Data sharing is not applicable to this article.
